# Patients With Inflammatory Bowel Disease Show IgG Immune Responses Towards Specific Intestinal Bacterial Genera

**DOI:** 10.3389/fimmu.2022.842911

**Published:** 2022-05-25

**Authors:** Arno R. Bourgonje, Geesje Roo-Brand, Paola Lisotto, Mehdi Sadaghian Sadabad, Rosanne D. Reitsema, Marcus C. de Goffau, Klaas Nico Faber, Gerard Dijkstra, Hermie J. M. Harmsen

**Affiliations:** ^1^ Department of Gastroenterology and Hepatology, University of Groningen, University Medical Center Groningen, Groningen, Netherlands; ^2^ Department of Medical Microbiology, University of Groningen, University Medical Center Groningen, Groningen, Netherlands; ^3^ Department of Rheumatology and Clinical Immunology, University of Groningen, University Medical Center Groningen, Groningen, Netherlands; ^4^ Department of Vascular Medicine, University of Amsterdam, Amsterdam University Medical Center, Amsterdam, Netherlands; ^5^ Wellcome Genome Campus, Wellcome Sanger Institute, Cambridge, United Kingdom

**Keywords:** inflammatory bowel disease, immune system, immunoglobulin G (IgG), microbiota, humoral immunity

## Abstract

**Introduction:**

Inflammatory bowel disease (IBD) is characterized by a disturbed gut microbiota composition. Patients with IBD have both elevated mucosal and serum levels of IgG-antibodies directed against bacterial antigens, including flagellins. In this study, we aimed to determine to which intestinal bacteria the humoral immune response is directed to in patients with IBD.

**Methods:**

Fecal and serum samples were collected from patients with IBD (*n*=55) and age- and sex-matched healthy controls (*n*=55). Fecal samples were incubated with autologous serum and IgG-coated fractions were isolated by magnetic-activated cell sorting (MACS) and its efficiency was assessed by flow cytometry. The bacterial composition of both untreated and IgG-coated fecal samples was determined by 16S rRNA-gene Illumina sequencing.

**Results:**

IgG-coated fecal samples were characterized by significantly lower microbial diversity compared to the fecal microbiome. Both in patients with IBD and controls, serum IgG responses were primarily directed to *Streptococcus*, *Lactobacillus*, *Lactococcus*, *Enterococcus*, *Veillonella* and *Enterobacteriaceae*, as well as against specific *Lachnospiraceae* bacteria, including *Coprococcus* and *Dorea* (all *P*<0.001), and to *Ruminococcus gnavus*-like bacteria (*P*<0.05). In contrast, serological IgG responses against typical commensal, anaerobic and colonic microbial species were rather low, e.g. to the *Lachnospiraceae* members *Roseburia* and *Blautia*, to *Faecalibacterium*, as well as to *Bacteroides*. Patients with IBD showed more IgG-coating of *Streptococcus*, *Lactobacillus*, and *Lactococcus* bacteria compared to healthy controls (all *P*<0.05). No differences in IgG-coated bacterial fractions were observed between Crohn’s disease and ulcerative colitis, between active or non-active disease, nor between different disease locations.

**Conclusion:**

The IgG immune response is specifically targeted at distinct intestinal bacterial genera that are typically associated with the small intestinal microbiota, whereas responses against more colonic-type commensals are lower, which was particularly the case for patients with IBD. These findings may be indicative of a strong immunological exposure to potentially pathogenic intestinal bacteria in concordance with relative immune tolerance against commensal bacteria.

## Introduction

Inflammatory bowel diseases (IBD), encompassing Crohn’s disease (CD) and ulcerative colitis (UC), are chronic ulcerative inflammatory diseases of the gastrointestinal (GI) tract, which are characterized by an inappropriate and uncontrolled immune response against microbiota in genetically susceptible individuals (1). The etiology of IBD remains incompletely understood, although it is believed that a complex interplay between genetic and environmental factors, the gut microbiota and host immunity is driving disease pathogenesis (2, 3). Gut microbiota alterations are commonly observed in patients with IBD, which are characterized by decreased microbial diversity, increased proportions of potentially pathogenic bacteria, and decreased proportions of commensal, butyrate-producing bacteria (4–6). Gut bacteria closely interact with the intestinal barrier, where a prodigious passage of luminal antigens occurs, which are continuously sampled by the underlying mucosal immune system as an immune surveillance mechanism (7). A compromised mucosal barrier is a hallmark of IBD, which results in excessive translocation of luminal antigens, eliciting both mucosal and systemic immune responses, which may in turn trigger and/or aggravate intestinal inflammation (8, 9).

Antimicrobial antibody responses are frequently observed in patients with IBD, particularly in CD (10, 11). Circulating IgG may leak into the intestinal lumen when mucosal barrier integrity is compromised by, for example, ulcerative inflammation (12, 13). This may result in increased IgG-coating of fecal bacteria. Indeed, we previously observed that IgG-coated fecal bacteria were overrepresented in CD compared with healthy controls, which may also associate with disease activity (13–15). Notably, patients with CD demonstrated elevated binding of IgG derived from autologous serum to fecal bacteria, suggesting earlier exposure to these bacterial antigens, perhaps during disease flares when mucosal barrier function was impaired (13). However, the exact antigenic nature of these IgG immune responses is largely unknown, as well as the exact bacteria that are targeted by autologous serum IgG.

In this study, we aimed to investigate to which bacterial genera the humoral IgG response is directed to in patients with IBD and in healthy individuals. To do so, we leveraged magnetic-activated cell sorting (MACS) and flow cytometry (FC) procedures to quantify the fractions of different IgG-coated bacteria after incubating fecal samples from patients and healthy individuals with their own (autologous) serum. Furthermore, we characterized the fecal microbial composition of both untreated and autologous serum-incubated fecal samples using 16S rRNA gene sequencing.

## Materials and Methods

### Study Population

Patients were included based on their participation in the String of Pearls initiative, a Dutch nation-wide project for which detailed phenotypic information and biological materials are collected from patients with IBD (16). Patients were recruited from the outpatient clinic of the department of gastroenterology in the University Medical Center Groningen (UMCG) and enrolled between 2010-2015. For this study, frozen fecal and serum samples were collected from patients with Crohn’s disease (CD) (*n*=34), ulcerative colitis (UC) (*n*=17) or inflammatory bowel disease unclassified (IBD-U) (*n*=4). For each patient, demographic and clinical information was collected, including age, sex, medication use (including biologicals), the Montreal disease classification, surgical history, and disease activity, all of which was assessed at time of sampling. Clinical disease activity was assessed using the Harvey-Bradshaw Index (HBI) for patients with CD and the Simple Clinical Colitis Activity Index (SCCAI) for patients with UC or IBD-U. The Montreal disease classification was recorded from their most recent visit to the outpatient clinic. Patients provided written informed consent for their participation in the study. This study was approved by the Institutional Review Board (IRB) of the UMCG (registered as no. 08/338) and was performed in accordance with the principles of the Declaration of Helsinki (2013). As controls, a cohort of 55 age- and gender-matched healthy individuals was included from the Northern Dutch Lifelines cohort study, from which only data on age and gender were recorded and who had self-proclaimed gastro-intestinal health. From these individuals, stool and serum samples were analyzed in a similar fashion as for those of patients with IBD.

### Sample Preparation

Fecal samples were diluted 50-fold by adding 0.25 grams of fecal sample to 12.25 ml filtered phosphate-buffered saline (PBS). Homogenized samples were centrifuged at 700 x*g* for 5 min to remove large particulates. The supernatant was stored in 1 ml aliquots at -20°C until further analysis. For 16S rRNA gene sequencing, DNA was extracted from fecal samples using the QIAamp DNA Stool Mini Kit (Qiagen, Hilden, Germany) according to the manufacturer’s instructions and following the exact same procedure as described previously (17). In addition, a bead beating cell lysis step was performed using a Precellys 24 homogenizer (Bertin Technologies, Aix-en-Provence, France) and glass beads (0.1 mm) at 5.5 m/s in three rounds of 1 min at room temperature.

### Magnetic-Activated Cell Sorting (MACS)

A 1 ml fecal sample aliquot (50-fold diluted) was centrifuged at 16,000 x*g* for 5 min, after which the bacterial pellet was dissolved in 1 ml PBS and centrifuged again. Subsequently, the bacterial pellet was resuspended in 100 μl autologous serum (100-fold diluted in PBS) and incubated on ice for 30 min. This suspension was added to 1 ml PBS/EDTA and centrifuged at 16,000 x*g* for 5 min. Next, the resulting bacterial pellet was resuspended in 500 μl Anti-Human IgG (γ-chain specific)-Biotin antibody (Sigma-Aldrich, St. Louis, MO, USA) which was 100-fold diluted in PBS/EDTA and incubated on ice for 30 min. Opsonized bacteria were sorted using a kit for magnetic-activated cell sorting (MACS) (Miltenyi Biotec GmbH, Bergisch Gladbach, Germany) as follows: 1 ml PBS/EDTA was added to the suspension and the mixture was subsequently centrifuged at 16,000 xg for 5 min. The bacterial pellet was suspended in 100 μl streptavidin-coated magnetic beads which was 10-fold diluted in PBS/EDTA and incubated on ice for 20 min, followed by another washing step with 1 ml PBS/EDTA and centrifugation at 16,000 x*g* for 5 min. Subsequently, the bacterial pellet was suspended in 500 μl PBS/EDTA. MS Columns were placed in the MACS Separator (magnetic plate) and were pre-rinsed with 500 μl PBS/EDTA. Next, 500 μl cell suspensions were applied onto a MS Column. Unlabeled cells were collected by washing the MS Columns three times with 500 μl PBS/EDTA. After this step, MS Columns were removed from the separator and placed on the new collection tube. In order to collect the magnetically labeled cells, 1.06 ml PBS/EDTA was applied onto the MS Column and immediately flushed out. The obtained liquids were stored at -20°C for DNA extraction and flow cytometry (FC) analysis.

### Flow Cytometry (FC)

A flow cytometry analysis was performed to confirm the efficiency of the MACS procedure. To do so, 50 μl of the magnetic bead-labeled fraction was resuspended in 100 μl Anti-Human IgG (γ-chain specific)-FITC antibody (Sigma-Aldrich, St. Louis, MO, USA) which was 100-fold diluted in PBS and incubated on ice in the dark for 30 min. This suspension was washed with 1 ml PBS and centrifuged at 16,000 x*g* for 5 min. The bacterial pellet was resuspended in 50 μl PBS while adding 10 μl propidium iodide (PI) (0.1 mg/ml) (Sigma-Aldrich, St. Louis, MO, USA). Fifty (50) μl of autologous serum-incubated stool supernatant suspension was labeled with Anti-Human IgG-FITC and another 50 μl was only incubated with PI as experimental controls. Samples were analyzed using the BD Accuri™ C6 flow cytometer (BD Biosciences, San Jose, CA, USA). Supernatant of three fecal samples with the largest bacterial pellet were washed with PBS and measured by flow cytometry to check the PI-induced background signal. The FL3-A signal above the threshold of these samples was measured in the analysis.

### Polymerase Chain Reaction (PCR) Amplification and 16S rRNA Gene Sequencing

Fecal microbial composition was determined by Illumina MiSeq paired-end sequencing of the V3-V4 hypervariable region of the 16S rRNA gene (MiSeq Bentchtop Sequencer, Illumina Inc., San Diego, USA). Amplification of bacterial DNA was performed by PCR using modified 341F and 806R primers with a six-nucleotide barcode on the 806R primer for multiplexing (18, 19). Both primers contain an Illumina MiSeq adapter sequence, which is necessary for flow cell binding in the MiSeq machine. Primer sequences can be found in **Supplementary Table S1**. A detailed description of the PCR amplification procedure, DNA clean-up and MiSeq library preparation using a 2x300 cartridge can be found in the **Supplementary Methods**. Demultiplexing and clustering of sequencing reads was performed using Quantitative Insights In Microbial Ecology (QIIME) with UCLUST v.1.2.22q at 97% similarity (20, 21). Taxonomic profiling was performed using Paired-eND Assembler for DNA sequences (PANDAseq) (22). All sequences with quality scores <0.9 were discarded by PANDAseq to increase sequence read-out quality, and read numbers per sample were rarefied to 25,000 read/sample. QIIME was used to assign bacterial taxonomy down to family and genus level, and ARB was used to identify sequences further down to species level (23).

### Statistical Analysis

Demographic and clinical characteristics of the study population were presented as means ± standard deviations (SD), medians with interquartile ranges (IQR) or proportions *n* with corresponding percentages (%). Differences between groups were analyzed using Mann-Whitney *U*-tests, while paired differences were determined used Wilcoxon signed-rank tests while adjusting for multiple comparisons under a false discovery rate (FDR) of 5%. Bacterial genera having a mean relative abundance above 0.25 were considered eligible for analysis. Microbial richness and diversity of samples was estimated with the Shannon diversity index as a measure of microbial alpha-diversity using QIIME. The microbial dissimilarities matrix (Bray-Curtis distances) was obtained using *vegdist* from the *vegan* R package and used as a measure of beta-diversity (24). Principal coordinates were constructed using the *cmdscale* function. Permutational multivariate analysis of variance (PERMANOVA) using distance matrices (ADONIS) was performed to analyze the variance in the Bray-Curtis dissimilarity matrix that could be explained by sample origin (IBD vs HC) or sample treatment (MACS-sorted samples vs. unsorted samples). Statistical analyses were performed using R (v.3.5.2) and the Python programming language (v.3.8.5, Python Software Foundation, https://www.python.org), using the *pandas* (v.1.2.3), *numpy* (v.1.20.0) and *scikit-bio* (v.0.2.3) modules. Data visualization was performed using the *seaborn* (v.0.11.1) and *matplotlib* (v.3.4.1) packages in Python. Two-tailed *P*-values ≤ 0.05 were considered as statistically significant.

## Results

### Cohort Characteristics

We included 55 patients with IBD (34 CD, 17 UC and 4 IBD-U) and 55 healthy controls (HC) (**Table 1**). Mean age was 44 (range: 20-64) years for patients with IBD and 46 (range: 20-64) years for HC. Among patients with IBD, 60% were female, which was comparable to 53% females among HC. Most patients with IBD were in clinical remission at the time of sampling (76%), whereas approximately one fourth of patients had active disease (24%).

**Table 1:** Demographic and clinical characteristics of the study population.

	IBD	HC	*P*-value
Age (years)	45 [32;54]	48 [36;59]	0.454
Sex, *n* (%)			0.442
*Male*	22 (40.0%)	26 (47.3%)	
*Female*	33 (60.0%)	29 (52.7%)	
IBD type, *n* (%)			
*CD*	34 (61.8%)	–	
*UC*	17 (30.9%)	–	
*IBD-U*	4 (7.2%)	–	
**Montreal classification**			
**Montreal Age (A)**			
A1 (≤ 16 years)	4 (7.2%)	–	
A2 (17-40 years)	27 (49.1%)	–	
A3 (> 40 years)	22 (40.0%)	–	
**Montreal Location (L), CD**			
L1 (ileal disease)	13 (38.2%)	–	
L2 (colonic disease)	11 (32.4%)	–	
L3 (ileocolonic disease)	10 (29.4%)	–	
**Montreal Behavior (B), CD**			
B1 (non-stricturing, non-penetrating)	23 (67.6%)	–	
B2 (stricturing)	7 (20.6%)	–	
B3 (penetrating)	4 (11.8%)	–	
**Montreal Perianal disease (P), CD**	3 (8.8%)	–	
**Montreal Extension (E), UC**			
E1 (proctitis)	3 (17.6%)	–	
E2 (left-sided colitis)	4 (23.5%)	–	
E3 (pancolitis)	10 (58.8%)	–	
**Montreal Severity (S), UC**			
S0 (remission)	4 (23.5%)	–	
S1 (mild)	4 (23.5%)	–	
S2 (moderate)	3 (17.6%)	–	
S3 (severe)	5 (29.4%)	–	
**Medication use**			
Aminosalicylates, *n* (%)	20 (36.3%)	-	
Steroids, *n* (%)	11 (20.0%)	-	
Immunosuppressives, *n* (%)	26 (47.2%)	-	
TNF-antagonists, *n* (%)	14 (25.5%)	-	
**Disease activity**			
Remission, *n* (%)	42 (76.4%)	-	
Active disease, *n* (%)	13 (23.6%)	-	
**Surgical history**			
Ileostomy/pouch, *n* (%)	7 (12.7%)	-	

Data are presented as proportions n with corresponding percentages (%) or as median [interquartile range, IQR] in case of continuous variables. CD, Crohn’s disease; HC, healthy control; IBD, inflammatory bowel disease; IBD-U, inflammatory bowel disease unclassified; TNF, tumor necrosis factor; UC, ulcerative colitis.

### Diversity of the Gut Microbiota in Original and IgG-Coated Fecal Samples

**Figure 1:**
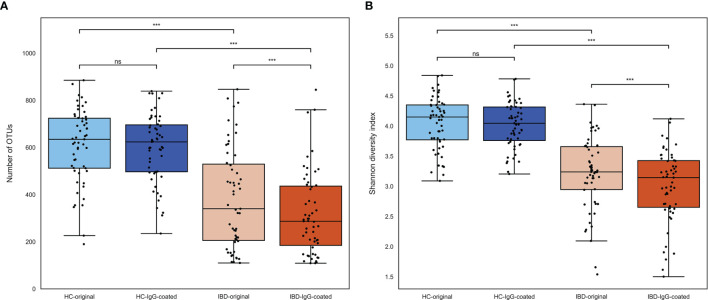
**(A, B)** Microbial richness and evenness of fecal samples from patients with IBD (orange colored) and HC (blue colored). **(A)** The number of OTUs was significantly lower in fecal samples from patients with IBD compared with samples from HC. OTUs were not significantly different after IgG-coating in samples from HC, whereas there was a small decrease in IgG-coated vs. untreated fecal samples from patients with IBD (paired analysis, *P* < 0.001). **(B)** The Shannon diversity index, representing the alpha-diversity of the gut microbiota, was significantly lower in patients with IBD vs. HC. Similar to the OTUs, IgG-coated vs. untreated samples showed no significant change in Shannon diversity index among HC, whereas there was a small decrease observed in IgG-coated samples from patients with IBD. HC, healthy controls; IBD, inflammatory bowel disease; MACS, magnetic-activated cell sorting; ns, non-significant; OTUs, operational taxonomic units. ***P < 0.001.

Microbial richness and diversity of both untreated (full fecal microbiome) and IgG-coated fecal samples are presented in **Figure 1**. The median number of operational taxonomic units (OTUs) per sample, representing sample richness of the gut microbiota, was significantly lower in samples of patients with IBD compared with samples from HC (298 [interquartile range [IQR]: 200-467] vs. 625 [IQR: 504-717], *P*<0.001) (**Figure 1A**). No significant paired difference in OTU counts were observed between original and IgG-coated fecal samples of HC (635 [IQR:512-724] vs. 624 [IQR: 497-696], *P*>0.05), whereas IgG-coated fecal samples from patients with IBD showed a decreased OTU count (340 [206-530] vs. 287 [185-436], *P*<0.001) compared with untreated fecal samples. The median Shannon diversity index, which represents the alpha-diversity (richness and diversity) of the gut microbiota, was significantly lower in IBD samples compared with samples from HC (3.2 [IQR: 2.7-3.5] vs. 4.1 [IQR: 3.8-4.3], *P*<0.001) (**Figure 1B**). Similar to the OTU counts, no paired difference was observed between untreated and IgG-coated fecal samples from HC (4.2 [IQR: 3.8-4.4] vs. 4.0 [3.8-4.3], *P*>0.05), whereas there was a small difference in untreated vs. IgG-coated samples from patients with IBD (3.2 [IQR: 2.9-3.7] vs. 3.1 [2.7-3.4], *P*<0.001).

**Figure 2:**
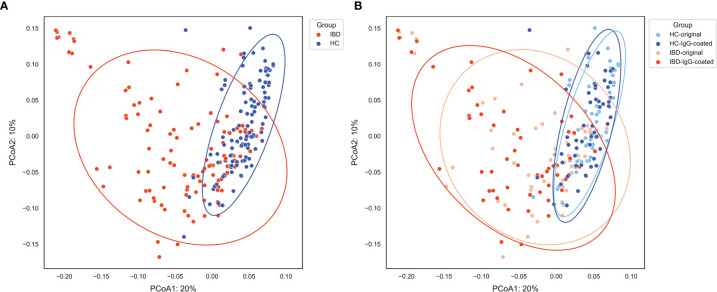
**(A, B)** Principal coordinate analysis (PCoA) of fecal samples from patients with IBD (orange) and HC (blue) visualizing the beta-diversity of the gut microbiota as represented by Bray-Curtis dissimilarities. **(A)** Sample origin (IBD vs. HC) shows an evident separation of the first two principal coordinates, which was statistically confirmed (PCoA1 and PCoA2: *P* < 0.001). **(B)** Paired analysis of IgG-coated vs. untreated fecal samples showed no significant change in the first two principal coordinates. Sample origin (IBD vs. HC) and sample handling (IgG-coated vs. untreated samples) significantly explained 20.5% and 2.2% of the variation in the gut microbiota, respectively. HC, healthy control; IBD, inflammatory bowel disease; MACS, magnetic-activated cell sorting; PCoA1, principal coordinate 1; PCoA2, principal coordinate 2.

The differences in microbial abundances between different fecal samples were evaluated by quantifying the beta-diversity of the gut microbiota, which was performed by calculating Bray-Curtis dissimilarity distances. The beta-diversity was significantly different between IBD samples and samples from HC in the first two principal coordinates (*P*<0.001), as can be observed from the principal coordinate analysis (PCoA) plots shown in **Figure 2**. Permutational multivariate analysis of variance using distance matrices (PERMANOVA, using the ADONIS function) demonstrated that sample origin (IBD vs. HC) was able to explain 20.5% of the variation in the beta-diversity of the gut microbiota (*P*=0.01). In contrast, beta-diversity was not significantly changed after the MACS procedure in the first two principal coordinates (both PCoA1 and PCoA2: *P*>0.05), which was confirmed after ADONIS analysis which revealed that only 2.2% of the variation (*P*=0.02) could be explained by sample handling (IgG-coating procedure vs. no coating). No significant associations between shifts (before and after IgG-coating) in the first four PCoAs *versus* clinical characteristics were observed (**Supplementary Figure S1**).

### Differences in Bacterial Composition Between Serum-Incubated IgG-Coated and Original Untreated Fecal Samples

**Figure 3:**
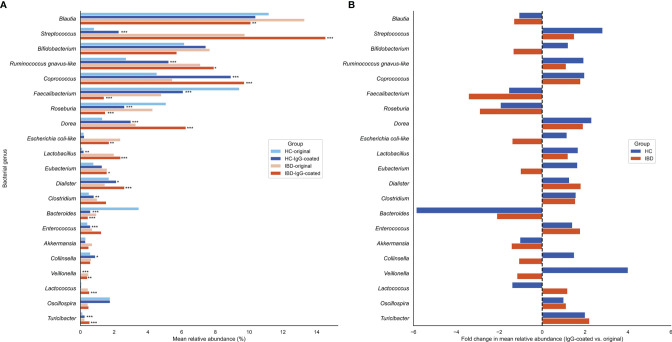
**(A, B)** Changes in relative abundances of fecal bacterial genera before and after IgG-coating in patients with IBD and HC. **(A)** Changes in mean relative abundances (%) after IgG-coating in fecal samples of patients with IBD (orange) and HC (blue). **(B)** Fold changes in mean relative abundances (%) before and after IgG-coating in IBD (orange) and HC (blue). Fold changes represent coating indices and were computed as follows: relative abundance (IgG-coated)/relative abundance (untreated). Relative increases in bacterial abundances are depicted on the right side of the plot, whereas decreases are displayed on the left side of the plot. Bacterial genera with relative abundances >0.25 were taken into statistical analysis. (^*^) indicate statistically significant increases or decreases in bacterial abundances: ^*^ *P* < 0.05; ^**^ *P* < 0.01; ^***^ *P* < 0.001. HC, healthy control; IBD, inflammatory bowel disease.

**Figure 4:**
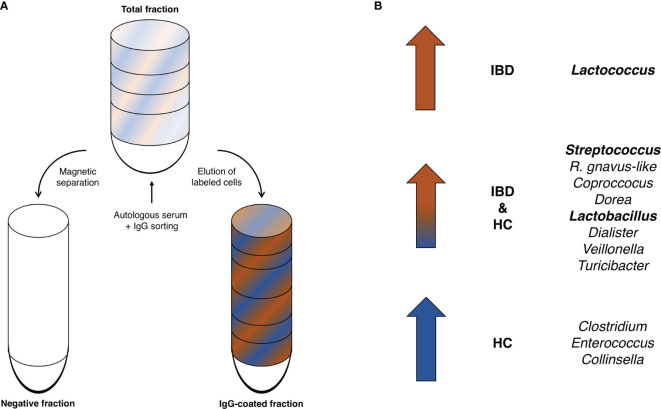
**(A, B)** Enriched IgG-coated fecal bacterial genera in patients with IBD and HC. **(A)** Schematic illustration of the IgG-based MACS-sorting procedure. The total fraction (top) represents the original, untreated sample, while the IgG-coated fraction represents the fraction left after magnetic separation of the non-IgG-coated bacterial fraction (negative fraction). **(B)** The bacterial genera *Streptococcus*, *Coprococcus, Dorea, Lactobacillus, Dialister, Veillonella, and Turicibacter as well as R. gnavus-*like bacteria were significantly enriched in IgG-coated samples in both patients with IBD and HC. *Lactococcus* bacteria were significantly enriched after IgG-coating solely in patients with IBD and *Clostridium*, *Enterococcus* and *Collinsella* solely in HC. Bold indicates bacterial genera that were significantly more enriched in IBD vs. HC.

The most common bacterial family observed in fecal samples was *Lachnospiraceae* (IBD: 40%; HC: 38%), followed by *Ruminococcaceae* (IBD: 14%; HC: 26%) and *Bifidobacteriaceae* (IBD: 6.7%; HC: 6.8%). The bacterial family of *Bacteroidaceae* accounted for a lower fraction of the microbial composition (IBD: 0.8%; HC: 2.0%). More variation in fecal microbial composition was observed on the genus level while comparing mean relative abundances (%) between IgG-coated vs. untreated fecal samples from patients with IBD and from HC (**Figure 3** and **Table 2**). Anti-IgG-based flow cytometry of fecal samples of patients with IBD revealed that between 13-77% (mean: 38%) of bacteria showed IgG-coating after incubation with autologous serum, which further increased to a range of 21-75% (mean: 45%) of enriched IgG-bound bacteria after MACS-sorting occurring in ~84% of samples (**Supplementary Table S2**). After the MACS procedure, several bacterial genera were significantly enriched in fecal samples from both patients with IBD and HC, including *Streptococcus*, *Coprococcus*, *Dorea*, *Ruminococcus gnavus*-like bacteria, *Lactobacillus*, *Dialister*, *Veillonella*, and *Turicibacter* (all *P*<0.05 in both groups). In total, 10 out of 21 (48%) bacterial genera demonstrated significant changes in abundance in IgG-coated vs. untreated fecal samples in both patients with IBD and healthy controls. The bacterial genera *Clostridium, Enterococcus*, and *Collinsella* were only significantly enriched in samples from HC, whereas *Lactococcus* was only significantly enriched in patients with IBD (**Figure 4**). In contrast to these enrichments, relative abundances of the bacterial genera *Faecalibacterium*, *Roseburia*, and *Bacteroides* were significantly decreased after IgG-coating in samples from both patients with IBD and HC (all *P*<0.05). In addition, the relative abundances of *Blautia*, *Eubacterium* and *Enterobacteriaceae-*like bacteria were solely decreased after IgG-coating in patients with IBD (*P*<0.01). Unpaired analysis between samples from patients with IBD and HC revealed that the bacterial genera *Streptococcus*, *Lactobacillus* and *Lactococcus* were more abundant in IBD (*P*<0.05).

**Table 2:** Paired analysis of mean bacterial abundances (>0.25) in original, untreated fecal samples versus IgG-coated fecal samples.

Genus	Family	HC			IBD		
		Average	Change	*P*-value^*^	Average	Change	*P*-value^*^
Blautia	Lachnospiraceae	11.17	-0.79	ns	13.28	-3.19	0.003
Streptococcus^#^	Streptococcaceae	0.81	+1.47	<0.001	9.74	+4.79	<0.001
Bifidobacterium	Bifidobacteriaceae	6.16	+1.27	ns	7.67	-1.96	ns
Ruminococcus gnavus-like	Lachnospiraceae	2.71	+2.52	<0.001	7.11	+0.80	0.035
Coprococcus	Lachnospiraceae	4.53	+4.39	<0.001	5.45	+4.26	<0.001
Faecalibacterium	Ruminococcaceae	9.42	-3.34	<0.001	4.80	-3.40	<0.001
Roseburia	Lachnospiraceae	5.07	-2.46	<0.001	4.28	-2.81	<0.001
Dorea	Lachnospiraceae	1.30	+1.69	<0.001	3.28	+2.97	<0.001
Escherichia coli-like	Enterobacteriaceae	0.20	+0.03	ns	2.37	-0.67	0.003
Lactobacillus^#^	Lactobacillaceae	0.12	+0.08	0.003	1.99	+0.39	<0.001
Eubacterium	Erysipelotrichaceae	0.78	+0.50	ns	1.58	-0.01	0.015
Dialister	Veillonellaceae	1.69	+0.44	0.02	1.45	+1.16	<0.001
Clostridium	Clostridiaceae	0.51	+0.29	0.008	0.99	+0.54	ns
Bacteroides	Bacteroidaceae	3.46	-2.87	<0.001	0.95	-0.54	<0.001
Enterococcus	Enterococcaceae	0.42	+0.17	<0.001	0.70	+0.54	ns
Akkermansia	Verrucomicrobiaceae	0.31	-0.01	ns	0.70	-0.21	ns
Collinsella	Coriobacteriaceae	0.59	+0.29	0.016	0.64	-0.05	ns
Veillonella	Veillonellaceae	<0.01	+0.03	<0.001	0.48	+0.07	0.003
Lactococcus^#^	Streptococcaceae	0.07	-0.02	ns	0.45	+0.08	<0.001
Oscillospira	Ruminococcaceae	1.76	-0.00	ns	0.44	+0.05	ns
Turicibacter	Erysipelotrichaceae	0.13	+0.13	<0.001	0.25	+0.30	<0.001

Data are presented as mean bacterial abundances (>0.25) sorted in descending order for patients with IBD. ^*^P-values for Wilcoxon’s signed rank tests. ^#^Bacterial genera that were differentially abundant between IBD and HC. HC, healthy controls; IBD, inflammatory bowel disease; ns, not significant.

## Discussion

In this study, we demonstrate that a variety of IgG immune responses are directed against the majority of the main fecal bacterial genera in both patients with IBD and healthy individuals. The IgG-coated samples showed a great overlap in enrichment of specific IgG-coated bacterial genera between both groups. In particular, serum IgG was directed against the bacterial genera *Streptococcus*, *Lactobacillus*, *Lactococcus*, and *Veillonella*. In contrast, bacterial genera like *Faecalibacterium*, *Roseburia*, and *Blautia* were decreased after the IgG-coating procedure, which are members of the families *Lachnospiraceae* and *Ruminococcaceae*, representing two dominant colonic microbial groups (**Figure 3**). This may suggest that there is less IgG reactivity against commensal (butyrate-producing) bacteria, however other *Lachnospiraceae* members, e.g. *Dorea* and *Coprococcus*, were significantly enriched after IgG-coating. This indicates that there were IgG immune responses against several types of commensal anaerobic microbiota. Furthermore, we showed that IgG-coating had a small but statistically significant impact on sample microbial diversity, which was to be expected as several types of bacteria were separated out after the MACS procedure. No significant differences in IgG-coated bacteria were observed between patients with CD and UC, patients with quiescent or active disease or patients with different disease locations, although numbers were actually too small to allow for reliable subgroup analyses. Collectively, these findings emphasize that both patients with IBD and HC have IgG immune responses against the fecal microbiota, many of which are rather prominent in patients with IBD. 

The observed IgG immune responses, particularly in patients with IBD, were preferentially directed against typical members of the small intestinal microbiota, including *Streptococcus*, *Lactobacillus*, *Lactococcus*, and *Veillonella*. These findings are in line with what is known on the composition of the small intestinal microbiota in the context of IBD. Bacteria that are typically enriched in the small intestine of patients with IBD as compared with population controls comprise, among others, *Streptococcus*, *Veillonella*, *Clostridium*, *Lactobacillus*, *Klebsiella*, *Enterococcus*, *Lactococcus*, and *Actinomyces* species (25–27). Moreover, some of these bacteria, especially *Streptococcus* and *Lactobacillus*, have also been found to be abundantly present within the mucosa-associated microbiota in inflamed biopsies from patients with IBD, residing within a thinner mucus layer compared to healthy controls (28). Considering these observations, one may speculate that a compromised small intestinal barrier integrity may lead to higher exposure of these bacteria to the mucosal immune system as they are in close proximity, resulting in increased specific IgG immune responses. However, this speculation was not sustained by our data as it can be anticipated that patients with CD, who often have small intestinal disease involvement, would exhibit increased IgG responses towards these bacteria compared to patients with UC, which was however not observed. In addition, our study was not sufficiently powered to reliably establish these potential differences between subtypes of IBD, and thus warrants further investigation. In keeping with this, the decreased IgG immune responses against commensal, anaerobic, butyrate-producing bacteria like *Roseburia* and *Faecalibacterium* may be explained by the fact that these bacteria confer barrier-protective and anti-inflammatory properties (29, 30). Butyrate is known for its anti-inflammatory and anti-carcinogenic effects, and it contributes to the preservation of the intestinal barrier by acting as an energy source for epithelial cells (30–32). Furthermore, these butyrate-producing bacteria are characterized by high oxygen sensitivity, which limits their passage of the intestinal barrier (as oxygen levels increase towards the intestinal mucosa), and, in turn, their potential exposure to the immune system (33). Of note, we observed a surprisingly low relative abundance and low IgG immune response against *Bacteroides*, whereas this genus is usually one of the most dominant bacterial groups detected in fecal samples (34). Therefore, the abundance of *Bacteroides* as observed in the present study (<1%) greatly underestimates its actual prevalence. One potential explanation for a relatively low IgG immune response could be that IgG responses to other bacterial genera were strongly enhanced, which could make the response to *Bacteroides* seem relatively lower since we have worked with *relative* bacterial quantifications. Another possible explanation could be that the specific primers that were used (341F/806R) may have failed to adequately amplify *Bacteroides*, but variations in sample storage conditions and DNA extraction procedures may also be responsible for such decreased abundance (35).

The observed enrichment after IgG-coating of several types of *Lachnospiraceae* (e.g. *Dorea* and *Coprococcus*) that belong to *Clostridium* cluster XIVa, but also that of many other bacteria like *Clostridium*, *Lactococcus*, *Lactobacillus*, and *Enterococcus* species could be explained by the fact that at least some species (e.g., *Enterocloster bolteae* and *Enterocloster clostridioforme*, or *Ruminococcus gnavus* belonging to the family of *Lachnospiraceae*) belonging to these bacterial genera are flagellated (10, 36, 37). Bacterial flagellae or flagellins are highly immunogenic proteins and dominant antigens in the context of IBD (36). Recently, a study demonstrated strong IgG immune responses against *Lachnospiraceae* flagellins in patients with CD, particularly those that were localized within the small intestine (11). This anti-flagellin antibody signature has been reported to be indicative of a more complicated disease course in CD, characterized by small intestinal disease involvement, frequent exacerbations and increased surgery rates (38, 39). Well-established flagellin antigens include CBir1, Fla-X, and A4-Fla2 (associated with *Lachnospiraceae* bacteria) (40, 41). Circulating anti-flagellin or antimicrobial antibodies are able to identify individuals who will develop CD years before the actual diagnosis, serving as serological predictors of the disease (42–45). A recent study showed that pre-existent anti-flagellin antibodies associated with future development of CD independently of subclinical inflammation, genetic susceptibility and intestinal barrier function (45). This suggests that the formation of adaptive immune responses against microbiota flagellins occurs very early in disease pathogenesis. Apart from the highly immunogenic properties of flagellins, bacterial flagella may facilitate transport across the epithelial mucus layer and enhance contact with a disrupted epithelial barrier, which may translate into an increased propensity to be exposed to the mucosal immune system (41, 46, 47).

An important observation of our study was that many of the enriched IgG-coated bacterial genera are consistently among those reported to be increased in abundance in patients with IBD (5, 6, 33). For example, this includes the previously mentioned (potentially pathogenic) small intestinal-type bacteria, but also *Enterobacteriaceae*, certain *Lachnospiraceae* (e.g., *Ruminococcus gnavus*), and *Clostridium* species. Similarly, patients with IBD consistently have lower numbers of *Faecalibacterium*, *Roseburia*, *Blautia* and *Bifidobacterium*, which were also not significantly enriched within the IgG-coated fractions in this study (48–50). Thus, bacterial groups that are commonly reported to be more abundant in patients with IBD compared with healthy individuals also largely overlap with those suggested to be more immunogenic based on findings from the present study.

Strengths of the present study include the fact that this study presents a unique characterization of antimicrobial immune responses against fecal bacteria in patients with IBD. Furthermore, the concurrent availability of an age- and sex-matched cohort of population controls enabled us to directly compare autologous IgG-coated fractions of fecal bacteria to determine their specificity for IBD. However, several limitations also warrant recognition. For instance, we could not confirm enrichment after IgG-coating for all fecal samples of patients with IBD (or we could not demonstrate this by flow cytometry), and the relative enrichment was also not that high when compared to earlier reports (51). One explanation to the limited enrichment observed in fecal samples of patients with IBD could be that anti-IgG-FITC is less effective when bacteria are already pre-treated with anti-IgG-biotin antibodies, which may in turn lead to underestimation of enrichment by anti-IgG-biotin/streptavidin-coated magnetic beads. In addition, the bacterial diversity in fecal samples from patients with IBD was significantly reduced after IgG-coating, suggesting that some degree of selection had occurred during the MACS-procedure, which might indicate the existence of selective immune responses towards specific bacteria in the context of IBD. Indeed, bacterial sequence results of the different sorted fractions demonstrated that there was a selection of specific bacterial groups. Another limitation to this study pertains to the relatively small sample size, which did not permit us to perform reliable subgroup analyses (e.g. a comparison between patients with CD and UC) as statistical power was very limited for this. In addition, we had to rely on clinical assessment of disease activity, as data on fecal calprotectin levels or endoscopic disease activity were not recorded at time of sampling. However, the majority of our cohort (~75%) was in disease remission and previous efforts already indicated that the degree of IgG-coating does not seem to be affected by disease activity (13).

In conclusion, this study demonstrates that patients with IBD exhibit distinct IgG immune responses directed towards a smaller group of specific types of bacteria compared with healthy controls. More specifically, this IgG immune response seems to be directed against *Streptococcus*, *Lactobacillus*, and *Lactococcus*, among others, as well as bacteria belonging to the family of *Lachnospiraceae* and other Clostridium cluster XIVa bacteria (e.g. *Coprococcus*, *Dorea*, and *Ruminococcus gnavus*-group). In contrast, there was some degree of IgG reactivity against typical colonic microbiota including several members of *Lachnospiraceae* (e.g., *Roseburia* and *Blautia*), *Ruminococcaceae* (e.g., *Faecalibacterium*), and *Bacteroides*, which was not clearly different between patients with IBD and HC. In addition, no significant differences in IgG reactivity were observed between CD and UC, quiescent and active disease or different disease locations. Future research is warranted to further unravel the pathophysiological role of these IgG-bound bacterial groups within the context of IBD. Further investigation into the exact antigenic nature of these specific bacteria is paramount to better characterize host-microbiota interactions that are relevant to IBD.

## Data Availability Statement

The raw sequencing data used for this study are publicly available via the NCBI BioProject repository under accession number PRJNA816096.

## Ethics Statement

The studies involving human participants were reviewed and approved by the Institutional Review Board (IRB) of the University Medical Center Groningen (full name in Dutch: “Medisch Ethische Toetsingscommissie”, METc). The patients/participants provided their written informed consent to participate in this study.

## Author Contributions

HH and GD were involved in conceptualization and study design. HH and GD were responsible for funding acquisition and resources. GR-B, MS, HM, and GD collected all study data. AB, GR-B, PL, MS, RR, MG, and HH performed data curation, data analysis, and visualization. AB, GR-B, and HH wrote the first draft of the manuscript. All authors contributed to results interpretation, critically reviewed the manuscript, contributed to manuscript revision, and read and approved the final version of the manuscript.

## Funding

The research position of AB was supported by a JSM M.D.-Ph.D. trajectory grant from the Junior Scientific Masterclass (JSM) of the University of Groningen, the Netherlands (grant number: 17-57). The funders had no role in the design of the study, collection, analysis, or interpretation of data, nor in writing of the manuscript.

## Conflict of Interest Statement

GD and HH received an unrestricted research grant from Royal DSM. GD received speaker fees from Pfizer, Abbvie and Janssen Pharmaceuticals.

The remaining authors declare that the research was conducted in the absence of any commercial or financial relationships that could be construed as a potential conflict of interest.

## Publisher’s Note

All claims expressed in this article are solely those of the authors and do not necessarily represent those of their affiliated organizations, or those of the publisher, the editors and the reviewers. Any product that may be evaluated in this article, or claim that may be made by its manufacturer, is not guaranteed or endorsed by the publisher.
