# Supplementary material for: Patients With Inflammatory Bowel Disease Show IgG Immune Responses Towards Specific Intestinal Bacterial Genera
Source: Front Immunol. 2022 May 25;13:842911. doi: 10.3389/fimmu.2022.842911 (PMC9174456; doi:10.3389/fimmu.2022.842911)
Supplement: Supplementary file 1 [file DataSheet_1.docx]

Supplementary Material

# Supplementary Methods

**Polymerase chain reaction (PCR), DNA clean-up, and MiSeq library preparation for 16S rRNA gene sequencing**

The PCR procedure consisted of the following conditions: an initial 94°C for 3 min followed by 32 cycles of 94°C for 45 sec, 50°C for 60 sec, and 72°C for 90 sec, with a final extension of 72°C for 10 min. Agarose gel electrophoresis confirmed the presence of the PCR product (band at ~465 bp) in successfully amplified samples. Subsequently, DNA samples were cleaned-up by thoroughly mixing the remainder of the PCR product with 25 μL Agencourt AMPure XP beads (Beckman Coulter, Brea, California, USA) followed by an incubation of 5 min at room temperature. Beads were separated from the mixture by placing the samples within a magnetic bead separator for 2 min. After discarding the cleared solution, beads were washed twice by resuspending them in 200 μL fresh 80% ethanol, followed by an incubation of 30 sec in the magnetic bead separator, and again discarding the cleared solution. The pellet was dried for 15 min and resuspended in 52.5 μL 10 mM Tris HCl buffer (pH 8.5). Fifty (50) μL of this solution was subsequently brought into a new tube. DNA concentrations were measured using Qubit® 2.0 fluorometer (Thermo-Fisher Scientific, Waltham, Massachusetts, USA) and the . To ensure similar library representations across samples, 2 nM dilutions of each sample were prepared accordingly. A library was created by pooling 5 μl of each diluted sample. Subsequently, 10 μL of the sample pool and 10 μL 0.2 M NaOH were mixed and incubated for 5 min to allow denaturation of the sample DNA. To this mixture, 980 μL of the HT1 buffer of the MiSeq 2x300 cartridge was added. Next, a denatured diluted PhiX solution was created by combining 2 μL 10 nM PhiX library with 3 μL 10 mM Tris HCl buffer (pH 8.5) with 0.1% Tween-20. To this mixture, 5 μL 0.2 M NaOH was added and incubated for 5 min at room temperature. This 10 μL-mixture was eventually mixed with 990 μL HT1 buffer. From the diluted sample pool, 150 μL was combined with 50 μL of the diluted PhiX solution, which was further diluted by the addition of 800 μL HT1 buffer. Finally, 600 μL of the prepared library solution was loaded into the sample loading reservoir of the 2x300 MiSeq cartridge for 16S rRNA amplicons sequencing (MiSeq Benchtop Sequencer, Illumina, San Diego, California, USA). Samples with low DNA concentrations after clean-up (quality score < 0.9) were discarded by PANDAseq to increase quality of sequence read-outs.

# Supplementary Tables

**Supplementary Table S1**. Nucleotide sequences of primers used for library construction for bacterial 16S rRNA gene (Illumina) sequencing.

| **Primer** | **Sequence** |
| --- | --- |
| V3_F_modified | aatgatacggcgaccaccgagatctacactctttccctacacgacgctcttccgatctNNNNCCTACGGGAGGCAGCAG |
| V4_1R | caagcagaagacggcatacgagat**CGTGAT**gtgactggagttcagacgtgtgctcttccgatctGGACTACHVGGGTWTCTAAT |
| V4_2R | caagcagaagacggcatacgagat**ACATCG**gtgactggagttcagacgtgtgctcttccgatctGGACTACHVGGGTWTCTAAT |
| V4_3R | caagcagaagacggcatacgagat**GCCTAA**gtgactggagttcagacgtgtgctcttccgatctGGACTACHVGGGTWTCTAAT |
| V4_XR | caagcagaagacggcatacgagat**XXXXXX**gtgactggagttcagacgtgtgctcttccgatctGGACTACHVGGGTWTCTAAT |

Bold uppercase letter highlight the index sequences (97 index sequences were used per sequence run, X indicates the subsequent index sequence), lower case letters indicate adapter sequences necessary for flow cell binding, underlined lowercase letters indicate binding sites for Illumina sequencing primers, and regular uppercase letters indicate the V3 and V4 region primers (341F for the forward primer, 806R for the reverse primer). The inclusion of four maximally degenerated bases (“NNNN”) maximizes the diversity during the first four bases of the run, which is important for unique cluster identification and base-calling accuracy.

**Supplementary Table S2**. Flow cytometry results after MACS enrichment of fecal samples of patients with IBD showing percentages of FITC-labeled bacteria enrichment after the MACS procedure.

| **Patient ID** | **Before MACS** | **After MACS** | **Enrichment** |
| --- | --- | --- | --- |
| 1 | 54% | 75% | +21% |
| 2 | 13% | 30% | +17% |
| 3 | 26% | 36% | +10% |
| 4 | 24% | 45% | +21% |
| 5 | 48% | 50% | +2% |
| 6 | 16% | 41% | +25% |
| 7 | 53% | 56% | +3% |
| 8 | 26% | 33% | +7% |
| 9 | 25% | 48% | +23% |
| 10 | 22% | 33% | +11% |
| 11 | 38% | 40% | +2% |
| 12 | 51% | 55% | +4% |
| 13 | 18% | 26% | +8% |
| 14 | 55% | 56% | +1% |
| 15 | 17% | 43% | 26% |
| 16 | 29% | 41% | +12% |
| 17 | 24% | 21% | -3% |
| 18 | 47% | 43% | -4% |
| 19 | 21% | 33% | +12% |
| 20 | 36% | 48% | +12% |
| 21 | 41% | 49% | +8% |
| 22 | 45% | 58% | +13% |
| 23 | 56% | 60% | +4% |
| 24 | 22% | 29% | +7% |
| 25 | 15% | 25% | +10% |
| 26 | 16% | 36% | +20% |
| 27 | 33% | 51% | +18% |
| 28 | 47% | 50% | +3% |
| 29 | 65% | 54% | -11% |
| 30 | 26% | 27% | +1% |
| 31 | 61% | 63% | +2% |
| 32 | 38% | 58% | +20% |
| 33 | 55% | 59% | +4% |
| 34 | 67% | 74% | +7% |
| 35 | 50% | 51% | +1% |
| 36 | 48% | 34% | -14% |
| 37 | 35% | 40% | +5% |
| 38 | 20% | 33% | +13% |
| 39 | 59% | 72% | +13% |
| 40 | 52% | 53% | +1% |
| 41 | 26% | 26% | 0 |
| 42 | 47% | 43% | -4% |
| 43 | 77% | 70% | -7% |
| 44 | 53% | 54% | +1% |
| 45 | 46% | 36% | -10% |
| 46 | 40% | 37% | -3% |
| 47 | 45% | 48% | +3% |
| 48 | 36% | 34% | -2% |
| 49 | 41% | 66% | +25% |
| 50 | 49% | 73% | +24% |
| 51 | 23% | 37% | +14% |
| 52 | 34% | 43% | +9% |
| 53 | 39% | 29% | -10% |
| 54 | 22% | 35% | +13% |
| 55 | 22% | 23% | +1% |
| Average | 38% | 45% | +7% |

# Supplementary Figures


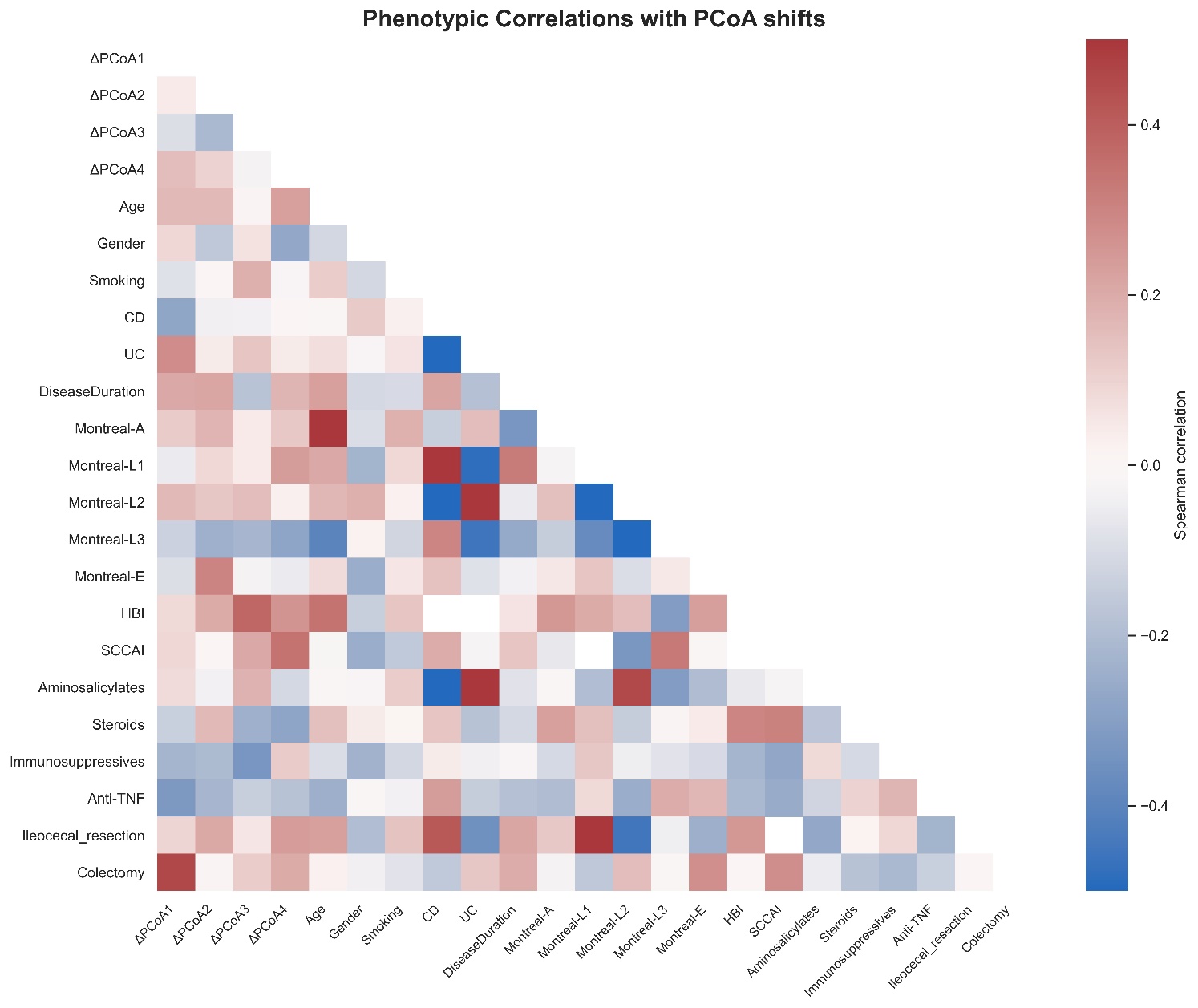


**Supplementary Figure S1**. Correlation triangle displaying correlations between clinical characteristics of patients with IBD and the main shifts in their microbial composition before and after the IgG-coating procedure. The first four principal coordinates (PCoAs, 1-4) were extracted from the microbial dissimilarity matrix (Bray-Curtis distances) and correlated with clinical characteristics. No statistically significant correlations were observed after adjustment for multiple comparisons.
